# RNF2 Mediates Hepatic Stellate Cells Activation by Regulating ERK/p38 Signaling Pathway in LX-2 Cells

**DOI:** 10.3389/fcell.2021.634902

**Published:** 2021-03-18

**Authors:** Qi Yan, Linxin Pan, Shunli Qi, Fang Liu, Zhen Wang, Cheng Qian, Lijian Chen, Jian Du

**Affiliations:** ^1^Department of Biochemistry and Molecular Biology, School of Basic Medical Sciences, Anhui Medical University, Hefei, China; ^2^The School of Life Science, Anhui Medical University, Hefei, China; ^3^Department of Anesthesiology, The First Affiliated Hospital of Anhui Medical University, Hefei, China; ^4^Center for Scientific Research, Anhui Medical University, Hefei, China

**Keywords:** inflammation, apoptosis, MAPK signaling pathway, RNF2, liver fibrosis, LX-2 cells

## Abstract

The therapeutic approach of liver fibrosis is still an unsolved clinical problem worldwide. Notably, the accumulation of extracellular matrix (ECM) in the liver is mediated by the production of cytokines and growth factors, such as transforming growth factor-β1 (TGF-β1) in hepatic stellate cells (HSCs). Ring finger protein 2 (RNF2) was identified as the catalytic subunit of polycomb repressive complex 1 (PRC1), mediating the monoubiquitination of histone H2A. In recent years, a growing amount of evidence suggests that RNF2 may play an important role in multiple pathological processes involved in cancer. Here, we explored the role of RNF2 in liver fibrogenesis and its potential mechanisms. The results showed that RNF2 was up-regulated in human fibrotic liver tissue. Knockdown of RNF2 led to a decreasing expression of collagen1 and α-smooth muscle actin (α-SMA) in LX-2 cells, which was upregulated by RNF2 overexpression. Moreover, RNF2 overexpression significantly promoted TGF-β1-induced LX-2 cell proliferation but decreased apoptosis. Furthermore, knockdown of RNF2 inhibited the activation of ERK/p38 signaling pathways induced by TGF-β1. These data suggested that RNF2 is an effective pro-fibrogenic factor for HSC activation via ERK/p38 signaling pathway. RNF2 inhibition might be a promising therapeutic target for liver fibrosis.

## Introduction

Liver fibrosis is a severe health problem worldwide resulting from chronic liver injury and viral hepatitis, comprising viral Hepatitis B (HBV) and hepatitis (HCV), alcoholic steatohepatitis (ASH), and non-alcoholic steatohepatitis (NASH), which may progress to cirrhosis, liver dysfunction, and even hepatocellular carcinoma (HCC) (Hernandez-Gea and Friedman, 2011; Sun and Kisseleva, 2015; Zoubek et al., 2017). Considerable evidence has revealed that the accumulation of excessive extracellular matrix (ECM) is a critical characteristic of liver fibrosis. The activation and proliferation of hepatic stellate cells (HSCs) may lead to the production of a large amount of ECM, which may result in an imbalance between synthesis and degradation, leading to liver fibrosis (Liu et al., 2019; Zhao et al., 2019). Recent studies have shown that HSCs are highly responsive to pro-inflammatory cytokines, and may exert specific effect on the regulation of inflammation during liver fibrosis (Seki and Schwabe, 2015). Hence, it is promising to find an intrinsic target for regulating inflammatory response and produce of ECM in HSCs. Therefore, targeting chronic inflammation under the circumstance of fibrogenesis will be a novel way to investigate the progress of liver fibrosis.

RING finger protein 2 (RNF2) is a member of the PcG (Polycomb group) family, which contains a conserved RING finger domain in its N-terminal region (Lee et al., 2005; Choi and Kang, 2011). Previous studies have shown that RNF2 was abnormally expressed in many types of cancer including HCC. Notably, down-regulation of RNF2 expression in HCC cells significantly reduced tumor cells growth and metastasis (Qu and Qu, 2017). In addition, RNF2 silencing inhibited the cell viability and resulted in increased G1 phase followed by a reduction of the G2/M phase (Zhang et al., 2017). Most HCC develops from liver inflammation, liver fibrosis and chronic liver injury. Of note, liver fibrosis is a necessary progress of various liver diseases (Baglieri et al., 2019). Therefore, it is essential to clarify the effect of RNF2 in the process of liver fibrosis.

Our experiment focused on the relationship between RNF2 and the process of liver fibrosis, especially in the regulation of HSCs. We chose LX-2 cells to research the effect of RNF2 in liver fibrosis, because the LX-2 cell line has main characteristics of HSCs, which are the central cells in the progress of liver fibrosis (Castilho-Fernandes et al., 2011). Our results showed that RNF2 exerts a pivotal effect in TGF-β1-induced LX-2 cells through the ERK/p38 signaling pathway.

## Materials and Methods

### Materials and Reagent

Fetal bovine serum was purchased from Biological Industries, and Dulbecco’s modified Eagle’s medium (DMEM) was purchased from HyClone. Lipofectamine^TM^3,000 and Trizol were purchased from Invitrogen. β-actin, RNF2, α-SMA, IL-6, COL 1, and TNF-α primers were purchased from Invitrogen. Recombinant TGF-β1 was purchased from Novoprotein Scientific (Shanghai). RNF2 (Cat:16031-1-AP) polyclonal antibody, β-actin monoclonal antibody, HRP-conjugated goat anti-rabbit IgG (SA00001-2), and goat anti-mouse IgG (SA00001-2) were purchased from Proteintech (China). pEGFP-C2-RNF2 plasmid was stored in our laboratory. PrimeScriptTM IV 1st Strand cDNA Synthesis Mix was bought from TaKaRa. FITC Annexin V apoptosis detection kit I (401007) was purchased from BestBio. Human IL-6 ELISA KIT (ml027379), human TNF-α ELISA KIT (ml077385) and human IL-1β ELISA KIT (ml058059) were purchased from Miblo (Shanghai China). TB Green^®^ Premix Ex Taq^TM^ II was purchased from TaKaRa. COL 1 (WL0088), α-SMA (WL02510) antibodies were purchased from Wanleibio (China). ECL chemiluminescent kit was purchased from NCM Biotech. BeyoClick^TM^ EdU Cell Proliferation Kit was purchased from Beyotime (Cat:C0075S).

### Specimen Collection

We collected human liver tissues (eight normal liver tissues and eight fibrotic liver tissues) in the First Affiliated Hospital of Anhui Medical University and performed further analyses. The normal liver samples were from patients with normal transaminase activity, no history of liver disease or alcohol abuse and no history of HBV or HCV infection. Additionally, the research complies with the “Declaration of Helsinki.” Meanwhile, it was approved by the Health Medical Research Ethics Committee of Anhui Medical University (20190214). All participants signed the patient’s informed consent form. The characteristics of patients and healthy donors are listed in Table 1.

**TABLE 1:** Characteristics of the subjects enrolled in the liver study.

Parameters	Healthy donor	Patients
**Case**, *n*	8	8
**Age**, *n*	49.375 (38–69)	57.375 (44–78)
**Sex**, *n* (%) male		6
	5 (62.5%)	6 (75%)
Female	3 (37.5%)	2 (25%)
**Etiology**, *n* (%) HBV		4 (50%)
HCV		0 (0%)
**HCC**, *n* (%)		
With		8 (100%)
Without		0 (0%)
**Serum ALT**, U/L	28.625	109
**Serum AST**, U/L	27	189.5

### Cell Culture and Stimulation

The LX-2 cells (human HSC line) were kindly donated by Professor Tao Xu, Anhui Medical University. Cells were cultured at 37°C in an atmosphere of 5% CO_2_, which were cultured in DMEM plus 10% FBS, 1% penicillin, and 1% streptomycin When LX-2 cells reached 70% confluence, they were treated with TGF-β1 (20 ng/ml) for 24 h.

### Cell Transfection and RNA Interference Analysis

LX-2 cells were inoculated in 6-well plate for 24 h before operation. Then the LX-2 cells were transfected with overexpression plasmid for RNF2 (pEGFP-C2-RNF2) using Lipofectamine^TM^3,000 (Invitrogen) according to the manufacturer’s protocol. On the other hand, LX-2 cells were infected with RNF2 knockdown lentivirus (termed as RNF2-shRNA), or a scramble control (termed as NC-shRNA), respectively, and then the positive cells were sorted by FCM (Beckman Coulter). Approximately 8,00,000 cells were seeded before transfection in six well plates. The mRNA and protein expression levels of RNF2 were measured at 48 h after transfection or infection. The pEGFP-C2-RNF2 and RNF2-shRNA concentrations were 1 μg/ml. The concentration and dose were listed in Table 2.

**TABLE 2:** Transfection and RNA interference.

	Concentration (μ g/μ l)	Dose (μ l)
pEGFP-C2-RNF2	1	2
pEGFP-C2	1	2
RNF2-shRNA	1	2
NC-shRNA	1	2

### RNA Extraction and Quantitative Real-Time PCR

Total RNA was extracted using TRIzol reagent and reverse transcribed into cDNA using PrimeScriptTM IV 1st Strand cDNA Synthesis Mix. RT-qPCR was performed by TB Green^®^ II RT-qPCR kit. Relative levels of specific mRNA were determined using the Roche LightCycler^®^ 96 RT-qPCR Detection System. Relative expression values were normalized using an internal β-actin control and we used the 2^–Δ^ ^Δ^ ^*CT*^ method to analyze the results. The primers sequences are listed in Table 3.

**TABLE 3:** Primer sequences for quantitative real-time reverse transcription polymerase chain reaction.

Gene	Primer pair	
	
α -SMA	F:5′-AGGCACCCCTGAAC CCCAA-3′	R:5′-CAGCACCGCCTGG ATAGCC-3′
COL 1	F:5′-CCCGGGTTTCAGAGAC AACTTC-3′	R:5′-TCCACATGCTTTATTCC AGCAATC-3′
β-actin	F:5′-GCCAACACAGTGCT GTCTGG-3′	R:5′-CTCAGGAGGAGCAAT GATCTTG-3′
IL-6	F:5′-ACTCACCTCTTCAGAAC GAATTG-3′	R:5′-CCATCTTTGGAAGGTT CAGGTTG-3′
TNF-α	F:5′-CCTCTCTCTAATCAGC CCTCTG-3′	R:5’-GAGGACCTGGGAGTA GATGAG-3′
IL-1β	F:5′-ATGATGGCTTATTACA GTGGCAA-3′	R:5′-GTCGGAGATTCGTA GCTGGA-3′

### Western Blotting

Proteins samples were extracted from human fibrotic and normal liver tissues and lysates of LX-2 cells by RIPA lysis buffer. The proteins of 30 μg per well were separated by SDS-PAGE. The primary antibodies recognizing RNF2, α-SMA, COL 1, ERK1/2, JNK, p38, p-ERK1/2, p-JNK, p-p38, and β-actin were used 1:1000, 1:500, 1:500, 1:500, 1:500, 1:500, 1:500, 1:500, 1:500, and 1:10000, respectively. HRP-conjugated secondary antibodies were used to incubated with membranes for 2 h at room temperature. The β-actin gene was used as an internal control for normalization. Quantitative intensity of the immunoblot images were performed by Image J v1.8.0 software.

### EdU Incorporation and Staining

Cell proliferation was detected by BeyoClick^TM^ EdU Cell Proliferation Kit. pEGFP-C2-RNF2 and RNF2-shRNA were, respectively, transfected and infected into LX-2 cells with Lipofectamine^TM^3,000. Then, 10 μmol/L EdU were added into cells for 2 h followed by washing with pre-cooling PBS. After marking, LX-2 cells were fixed and permeabilized. Click Additive Solution was incubated for 30 min at room temperature. The images were taken by fluorescence microscopy.

### Flow Cytometry

Annexin V-PE/7-AAD apoptosis kit was chosen for detecting cell apoptosis. LX-2 cells were transfected or infected with RNF2 (pEGFP-C2-RNF2 or RNF2-shRNA) at 37°C for 24 h. After washing with pre-cooling PBS, the cells were resuspended in 100 μl 1× binding buffer. Subsequently, 5 μl of Annexin V-PE and 5 μl of 7-AAD were added to each tube and mixed. Then they were incubated at room temperature for 15 min, and 400 μl 1× binding buffer was added. The flow cytometer was used to detect the apoptosis.

### ELISA Assay

The supernatants of LX-2 cells were used to detect the expression levels of proinflammatory cytokine TNF-α, IL-1β, and IL-6, all analyses were performed according to instructions provided by the kit manufacturer.

### Immunohistochemical Staining

Immunohistochemical staining was used to detect expression levels of α-SMA and RNF2 in normal and fibrotic liver tissues. Briefly, liver sections were deparaffinized rehydrated, and antigen was repaired. The sections were blocked by 5% BSA, then primary antibodies against rabbit RNF2 and α-SMA were incubated overnight at 4°C. The secondary antibodies were incubated for 1 h at room temperature. The diaminobenzidine was used to visualize the immune reaction.

### Statistical Analysis

Assays were conducted at least in triplicate independently. The differences in groups were checked by one-way analysis of variance (ANOVA). All experimental results were performed by SPSS 18.0. Data are expressed as mean ± standard deviation of the mean (SD). *P* < 0.05 indicated a statistically significant difference, and *P* < 0.01 indicated a strongly significant difference.

## Results

### RNF2 Is Upregulated in Fibrotic Liver Tissues and LX-2 Cells Induced by TGF-β1

**FIGURE 1:**
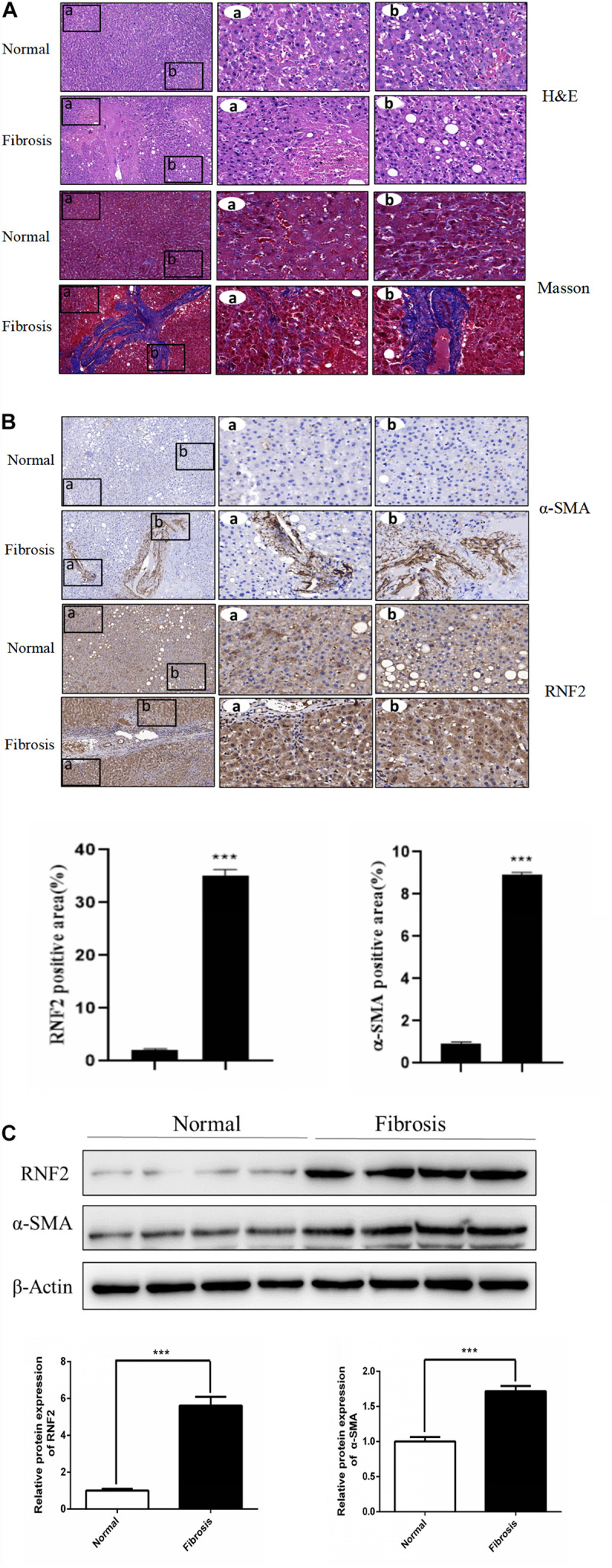
RNF2 was increased in human fibrotic liver tissues. **(A)** The H&E and Masson staining result. **(B)** The immunohistochemistry of RNF2, α-SMA in human fibrotic liver tissues and normal tissues. **(C)** The protein expression level of RNF2, α-SMA in human fibrotic liver tissues and normal tissues. ***P < 0.001 compared with the normal group.

**FIGURE 2:**
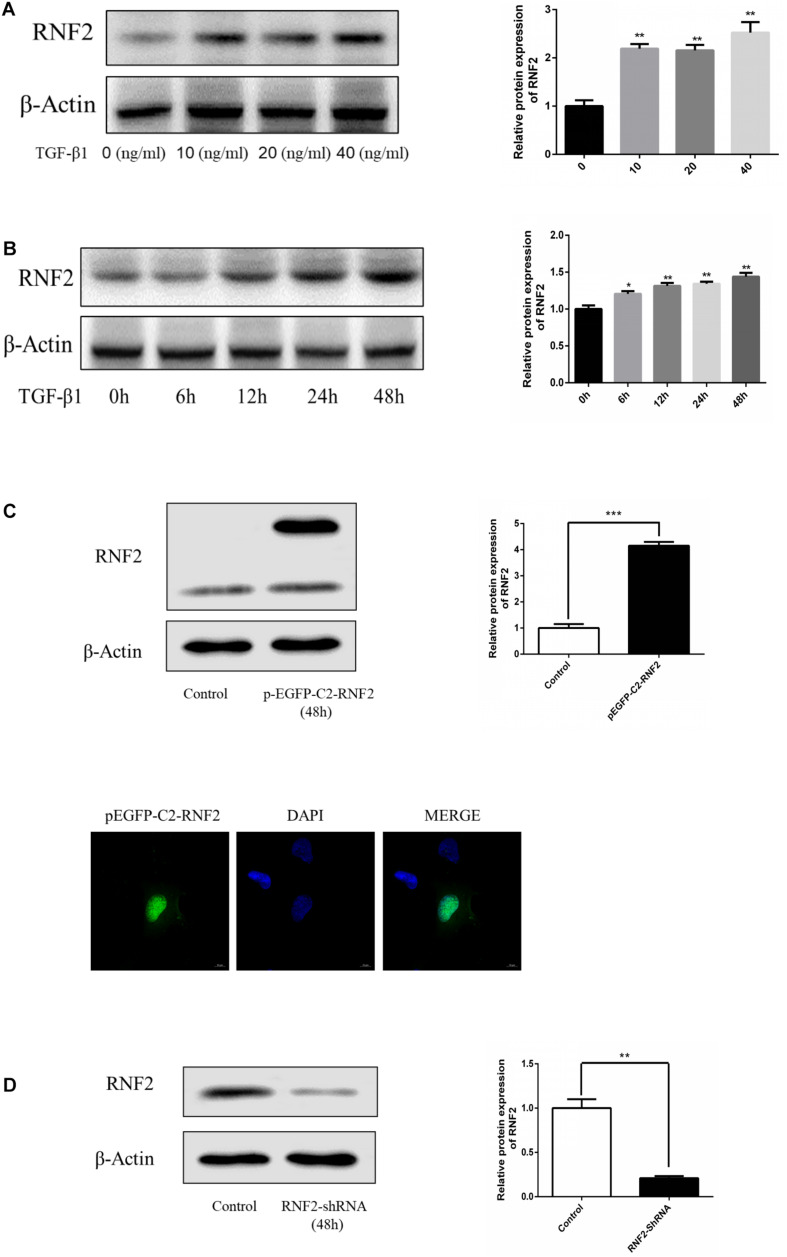
TGF-β1 induced the expression of RNF2 in LX-2 cells. **(A)** TGF-β1 dose-dependent expression of RNF2 in LX-2 cells. **(B)** TGF-β1 time-dependent expression of RNF2 in LX-2 cells. **(C)** Lipidosome-mediated transfection with pEGFP-C2-RNF2 plasmid resulted in RNF2 overexpression in LX-2 cells. Immunofluorescence assay was performed on LX-2 cells transfected with pEGFP-C2-RNF2, and the result showed that RNF2 protein was mainly located in the nucleus. **(D)** Lipidosome-mediated transfection with RNF2-shRNA lentivirus resulted in RNF2 knockdown in LX-2 cells. *P < 0.05 compared to the normal group. **P < 0.01 compared to the normal group. ***P < 0.001 compared to the normal group.

We first detected whether the expression of RNF2 in human fibrotic liver tissues has changed. The results of H&E staining and Masson staining showed that human fibrotic liver tissues have more severe steatosis and necrosis than the normal group (Figure 1A). Immunohistochemistry results suggested that the one important marker of liver fibrosis (α-SMA) was obviously up-regulated in fibrotic tissues (Figure 1B). Notably, Western blotting results showed that the expression of RNF2 in human fibrotic liver tissue is significantly higher than that in normal liver tissue (Figure 1C). The same result was confirmed by immunohistochemistry (Figure 1B). Besides, the expression level of RNF2 was observed at different concentrations and times with TGF-β1 stimulation in LX-2 cells. Western blotting results indicated that the noteworthy upregulation protein level of RNF2 were observed at 20 ng/mL in TGF-β1-induced LX-2 cells (Figure 2A). And RNF2 expression was obviously increased when TGF-β1 stimulated LX-2 cells for 24 h (Figure 2B). According to the results, we could speculate that RNF2 might participate in the liver fibrogenesis, especially in activating HSCs.

### Effect of RNF2 on Activation of HSCs in LX-2 Cells Induced by TGF-β1

**FIGURE 3:**
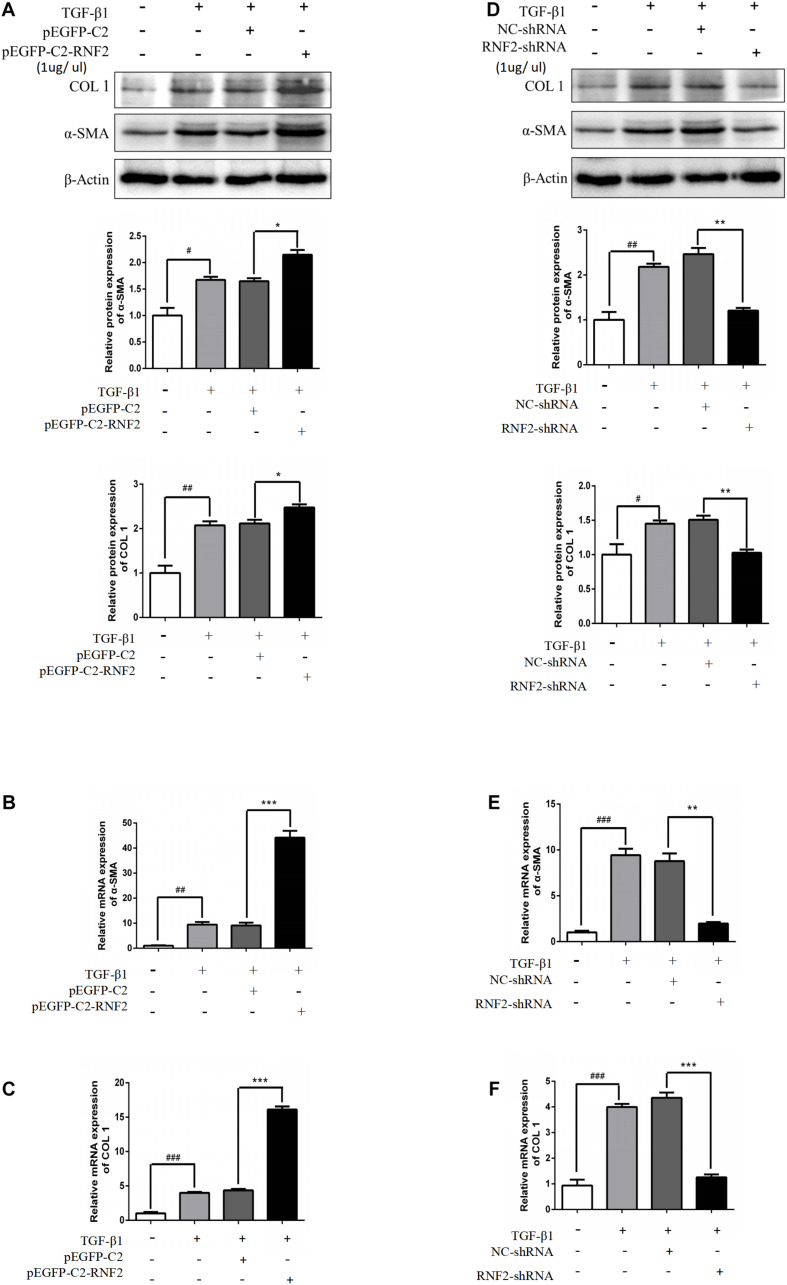
RNF2 regulated LX-2 cells activation positively. **(A–C)** The α-SMA and COL 1 expression levels were measured by Western Blotting and RT-qPCR in activated LX-2 cells transfected with pEGFP-C2-RNF2 (1 μg/μl) and pEGFP-C2 (1 μg/μl) plasmid, respectively. The results showed that, compared to TGF-β1 group, there was a nearly four-fold increase on the relative α-SMA and COL1 mRNA levels of LX-2 transfected with pEGFP-C2-RNF2. **(D–F)** The α-SMA and COL 1 expression levels were measured by Western Blotting and RT-qPCR in activated LX-2 cells infected with RNF2-shRNA (1 μg/μl) and NC-shRNA (1 μg/μl) lentivirus, respectively. The results showed that, compared to TGF-β1 group, there was a nearly four-fold decrease on the relative α-SMA mRNA levels and a five-fold decrease on the relative COL1 mRNA levels of LX-2 transfected with RNF2-shRNA. The change is almost negligible between TGF-β1 group and NC-shRNA group. ^#^*P* < 0.05, ^##^*P* < 0.01, and ^###^*P* < 0.001 compared to the normal group *P < 0.05, **P < 0.01, and ***P < 0.001 compared to the TGF-β1 group.

Next, pEGFP-C2-RNF2, and RNF2-shRNA lentivirus were, respectively, transfected and infected into LX-2 cells to increase and decrease the RNF2 expression (Figures 2C,D). The results showed that pEGFP-C2-RNF2 obviously promoted COL 1 and α-SMA expression (Figures 3A–C). In addition, RNF2-shRNA inhibited the expression levels of COL 1 and α-SMA (Figures 3D–F). Taken together, these results demonstrated that RNF2 was positively related to HSCs activation.

### Effect of RNF2 on IL-1β IL-6, and TNF-α Secretion in LX-2 Cells Induced by TGF-β1

**FIGURE 4:**
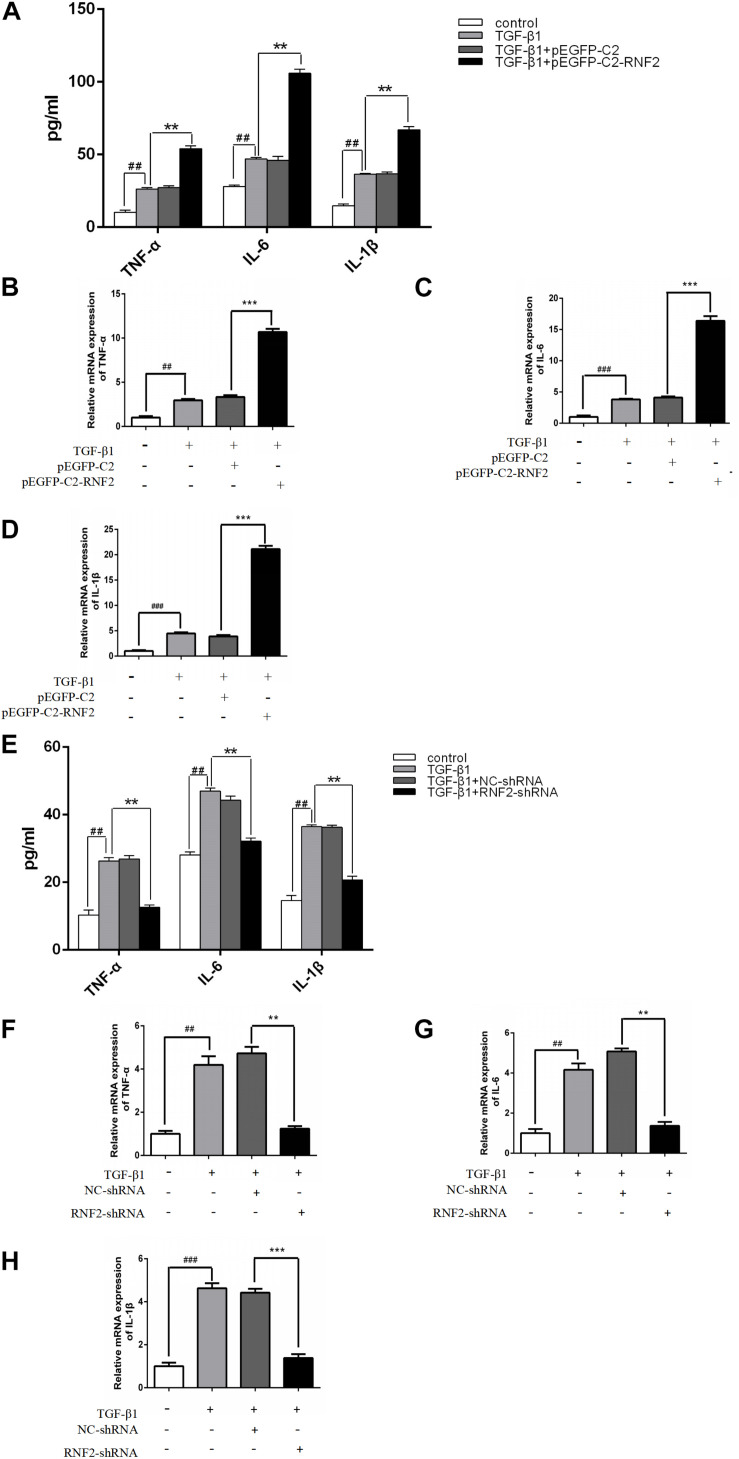
Effect of RNF2 on the secretion of TNF-α, IL-6 and IL-1β in activated LX-2 cells. **(A–D)** The expression levels of TNF-α, IL-6, and IL-1β were measured by ELISA and RT-qPCR in LX-2 cells transfected with EGFP-C2-RNF2. The results showed that the relative TNFα, IL-6, and IL-1β mRNA levels of LX-2 transfected with PEGFP-C2-RNF2 increased approximately with three-fold, four-fold, and five-fold, respectively, compared to TGF-β1 group. The results are expressed as the mean ± SD of three different experiments. ^##^*P* < 0.01 and ^###^*P* < 0.001 compared to the normal group, **P < 0.01 and ***P < 0.001 compared to the TGF-β1 group. **(E–H)** The expression levels of TNF-α, IL-6, and IL-1β were measured by ELISA and RT-qPCR in LX-2 cells infected with RNF2-shRNA lentivirus. The results showed that, the relative TNFα, IL-6, and IL-1β mRNA levels of LX-2 transfected with RNF2-shRNA decreased approximately with three-fold compared to TGF-β1 group. The change is almost negligible between TGF-β1 group and NC-shRNA group.

In order to clarify whether RNF2 is involved in the secretion of inflammatory cytokines in LX-2 cells, we transfected or infected pEGFP-C2-RNF2 or RNF2-shRNA with LX-2 cells, respectively, before induction of TGF-β1. The results showed that TGF-β1 significantly increased the mRNA expression levels of TNF-α, IL-1β, and IL-6 (Figures 4B–D). On the contrary, the phenomenon has been reversed by knockdown RNF2 (Figures 4F–H). In addition, pEGFP-C2-RNF2 significantly upregulated TNF-α, IL-1β, and IL-6 secretion induced by TGF-β1 (Figure 4A), knockdown of RNF2 had the opposite effect (Figure 4E). ELISA assay results indicated that pEGFP-C2-RNF2 had a positive effect on TNF-α, IL-1β, and IL-6 secretion. Hence, we demonstrated that RNF2 could lead to the increase in the expression of IL-1β, IL-6, and TNF-α in LX-2 cells induced by TGF-β1.

### Effect of RNF2 on HSCs Proliferation and Apoptosis in LX-2 Cells Induced by TGF-β1

**FIGURE 5:**
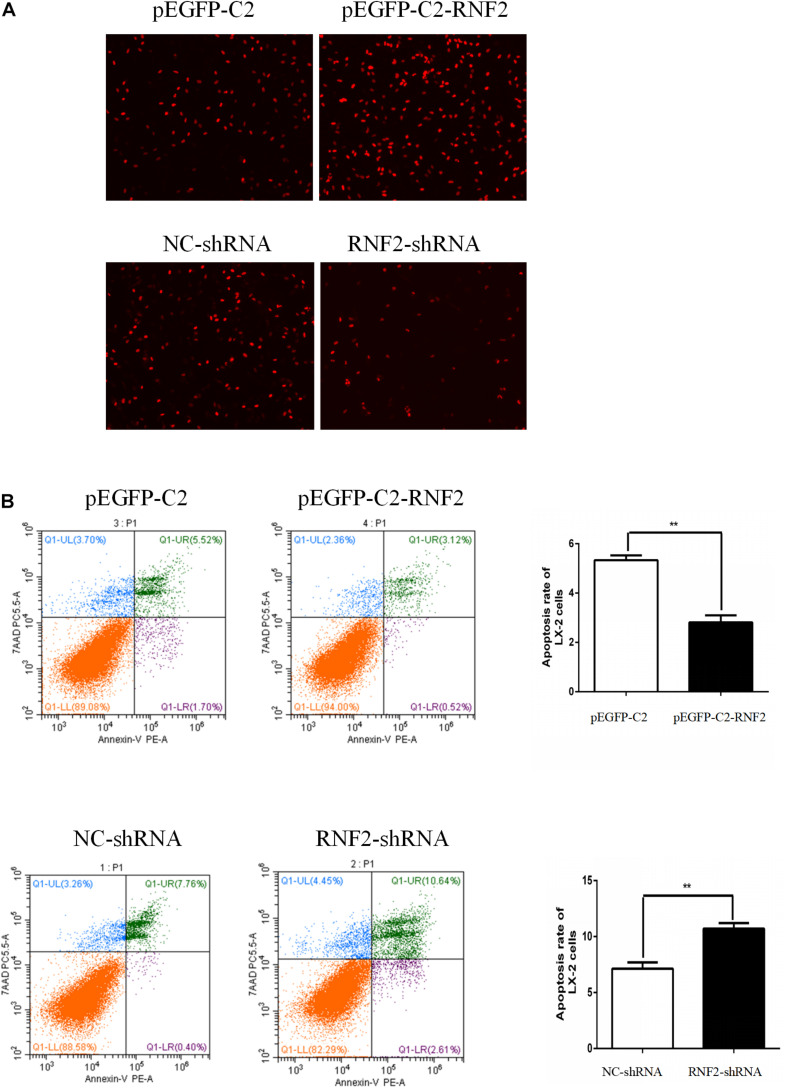
Effect of RNF2 on cell proliferation and apoptosis in TGF-β1-stimulated LX-2 cells. **(A)** Proliferation of LX-2 cells was determined by EdU DNA incorporation assay. **(B)** Cell apoptosis of LX-2 cells was measured by flow cytometry analysis.

EdU staining was used to discover the effect of RNF2 on cell proliferation. The results indicated that pEGFP-C2-RNF2 promoted cell proliferation in activated LX-2 cells, while RNF2-shRNA had the opposite effect (Figure 5A). In addition, the flow cytometry results demonstrated that pEGFP-C2-RNF2 significantly inhibited cell apoptosis in LX-2 cells and RNF2 silencing showed a completely different phenomenon (Figure 5B). In general, these results proved that RNF2 could inhibit cells apoptosis in TGF-β1-induced LX-2 cells.

### Effect of RNF2 on MAPK Signaling Pathway in LX-2 Cells Induced by TGF-β1

**FIGURE 6:**
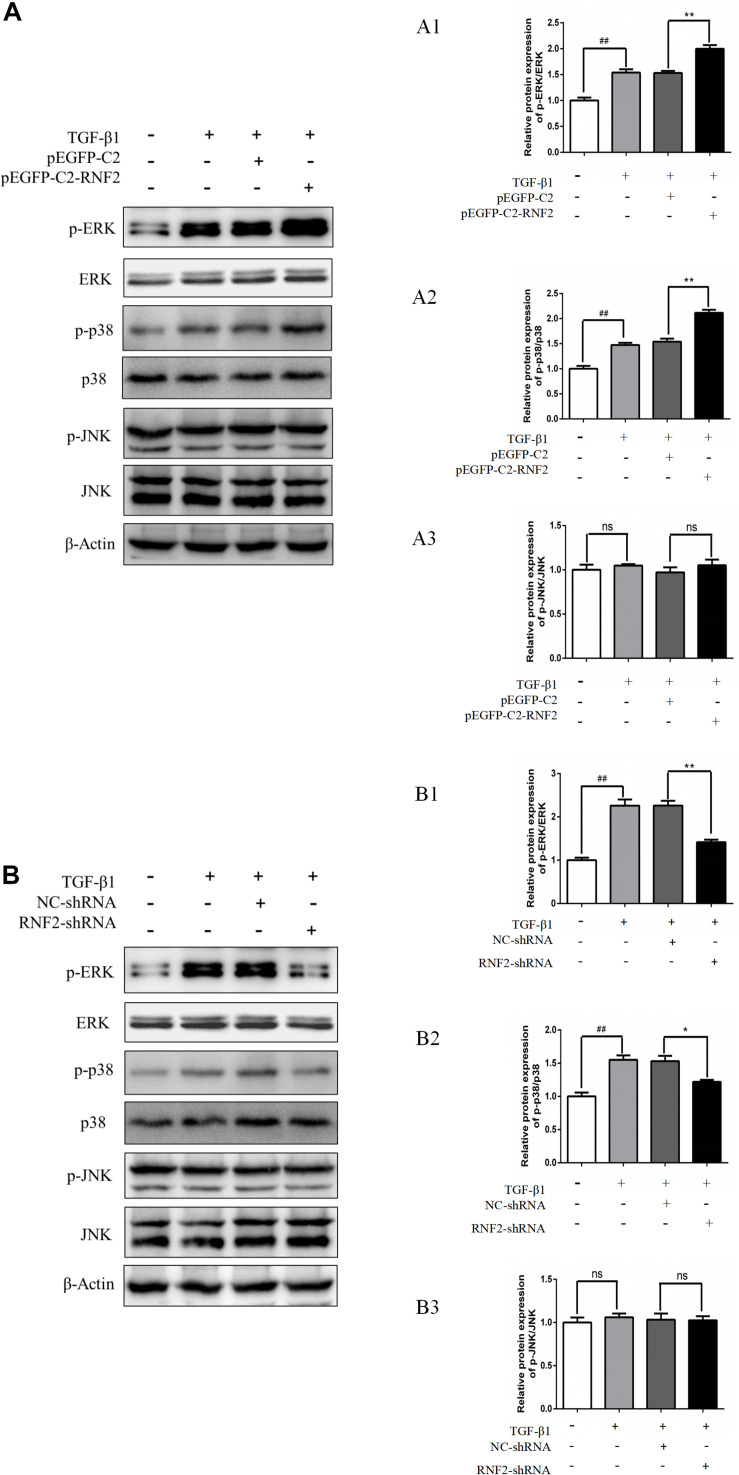
Effect of RNF2 on MAPK signaling pathway in TGF-β1-stimulated LX-2 cells. **(A)** LX-2 cells were stimulated by TGF-β1 for 24 h after transfected with pEGFP-C2-RNF2 or pEGFP-C2 plasmid. Western blotting was used to detect associated proteins of MAPK signaling pathway. **(B)** LX-2 cells were stimulated by TGF-β1 for 24 h after infected with RNF2-shRNA or NC-shRNA lentivirus. Western blotting was used to detect associated proteins of MAPK signaling pathway. ^#^*P* < 0.05, ^##^*P* < 0.01, and ^###^*P* < 0.001 compared with control, **P < 0.01 and ***P < 0.001 compared with LX-2 cells treated with TGF-β1. ns suggest they have no statistical differences. The results demonstrated that RNF2 overexpression activated ERK/p38 signaling pathway, and RNF2 silencing showed the opposite effect. However, there is no change in expression of p-JNK after the change of RNF2.

Previous studies have shown that the MAPK signaling pathway was closely related to the activation of HSCs (Zhang et al., 2019). Furthermore, the associated proteins of the signaling pathway were detected. The results indicated that the expression level of p-p38/p-ERK was increased after transfecting with pEGFP-C2-RNF2 (Figure 6A). However, there is no change in the expression of p-JNK after the change of RNF2. In addition, low expression of RNF2 inhibited the protein expression levels of p-p38/p-ERK (Figure 6B). In a word, these data suggested that RNF2 regulates the development process of liver fibrosis via ERK/p38 signaling pathway.

## Discussion

Liver fibrosis is a reversible pathological process, leading to progressive accumulation of ECM. As we all known, activation of HSCs is regarded as a critical step in the development of liver fibrosis (Foglia et al., 2019). The HSC is an important fibrogenic cell type in the liver, and is involved in the deposition of ECM of the liver (Wells, 2008). During liver injury, HSCs activation is observed, accompanied by up-regulation of α-SMA expression (activated HSC marker) (He et al., 2012). Liver fibrosis is a pathophysiological process. Of note, long-term persistent liver fibrosis will develop into HCC (Toosi, 2015). Most HCC develops in the context of liver fibrosis, during which HSCs transition from a quiescent to an activated state (Chen et al., 2020). Accumulating studies have demonstrated that loss of RNF2 inhibited HCC cell proliferation and promoted apoptosis (Qu and Qu, 2017). However, the relationship between liver fibrosis and RNF2 remains to be further explored.

In our study, the data firstly demonstrated that RNF2 expression significantly up-regulated in human fibrotic liver tissues by Western blotting and immunohistochemistry. In addition, RNF2 expression was significantly upregulated in TGF-β1-stimulated LX-2 cells. As we all know, TGF-β1 is the most potent cytokine responsible for the regulation of the HSC phenotype. Notably, we found that RNF2 could increase the expression level of α-SMA and COL 1 both at mRNA and protein levels in TGF-β1-induced LX-2 cells, suggesting that RNF2 regulated HSCs activation positively. Notably, severe inflammation may generate and exacerbate liver injury and fibrosis (Koyama and Brenner, 2017). This research also indicated that the expression of TNF-α, IL-1β, and IL-6 were significantly increased in TGF-β1-induced LX-2 cells. Overexpression of RNF2 might increase the expression level of IL-6, TNF-α, and IL-1β in TGF-β1-stimulated LX-2 cells. Conversely, RNF2 silencing could decrease the expression level of TNF-α, IL-1β, and IL-6. Based on the data, we can draw the novel conclusion that RNF2 plays a key role in inflammatory cytokine secretion, such as TNF-α, IL-1β, and IL-6. In the aspect of cell activity, we used cell proliferation as an important mechanisms indicator to determine cell viability (Adan et al., 2016). EdU DNA incorporation assay was used to detect the proliferation of HSCs, which suggested that RNF2 could observably promote the proliferation of in TGF-β1-stimulated LX-2 cells. Moreover, the results of flow cytometry indicated that HSCs apoptosis was downregulated by RNF2 in TGF-β1-induced LX-2 cells.

Mechanically, MAPK signaling pathway is associated with liver fibrosis. As is known, TGF-β1 is an important activating factor of MAPK signaling pathway (Gao et al., 2016; Yang et al., 2018). It is shown that abnormal MAPK signaling is involved in the development of organ fibrosis including liver fibrosis. Indeed, MAPK signaling pathway is critical for HSCs activation. Moreover, liver fibrosis was found to be reversible (Ellis and Mann, 2012; Atta, 2015). Despite increased understanding of RNF2 function, some aspects related to mechanisms of sensing and downstream signaling remain elusive. Previous research showed that RNF2 may participate in the progress of MAPK signaling pathway activation (Rao et al., 2009), but the relationship between RNF2 and MAPK signaling pathway remains to be further examined in HSCs. Indeed, we found that TGF-β1-stimulated phosphorylation of p38 and ERK level in LX-2 cells was inhibited in LX-2 cells pretreated with RNF2-shRNA. In addition, RNF2 overexpression significantly increased the p-ERK and p-p38 expression in TGF-β1-stimulated LX-2 cells. However, no detectable activation of p-JNK could be observed. Taken together, these results demonstrated that RNF2 exerts its promoting effect on HSC activation via regulating the ERK/p38 signaling pathway positively.

In brief, the latest data indicated that RNF2 was crucial for the progression of HSCs activation regulated by ERK/p38 signaling pathway. It is generally accepted that knockout mice and the features of RNF2 in liver inflammatory injury will be considered as the next research highlight. Based on the outstanding role of RNF2, finding new target drugs for liver disease may be subsequent in the future. Therefore, understanding the roles of RNF2 in cell proliferation, inflammation, and further functions during liver fibrosis even other liver diseases is useful work, which brings a potentially therapeutic approach for the treatment of liver disease.

## Data Availability Statement

The original contributions presented in the study are included in the article/supplementary material, further inquiries can be directed to the corresponding author/s.

## Ethics Statement

The studies involving human participants were reviewed and approved by Health Medical Research Ethics Committee of Anhui Medical University (20190214). The patients/participants provided their written informed consent to participate in this study.

## Author Contributions

LC and JD designed the study. QY and LP participated in the collecting and analyzing of the data. SQ and FL finished the manuscript. ZW and CQ revised and edited the manuscript. All authors approved the final version of the manuscript for publication.

## Conflict of Interest

The authors declare that the research was conducted in the absence of any commercial or financial relationships that could be construed as a potential conflict of interest.
